# A method to induce stress in human subjects in online research environments

**DOI:** 10.3758/s13428-022-01915-3

**Published:** 2022-07-25

**Authors:** Mohammed A. Almazrouei, Ruth M. Morgan, Itiel E. Dror

**Affiliations:** 1 UCL Department of Security and Crime Science, University College London, 35 Tavistock Square, London, WC1H 9EZ UK; 2 UCL Centre for the Forensic Sciences, University College London, 35 Tavistock Square, London, WC1H 9EZ UK; 3 Forensic Evidence Department, Abu Dhabi Police General Headquarters, Abu Dhabi, 253 UAE

**Keywords:** Online study, Stress, Human subjects, COVID-19, Crowdsourcing

## Abstract

This paper presents a method to induce stress in human subjects during online participation in research studies without the presence of researchers. In this study, participants in the stress-inducing condition (*N* = 52, 44%) were asked to answer general knowledge and mathematical questions which people often get wrong, and did so under time pressure as well as receiving feedback. In contrast, participants in the control condition (*N* = 66, 56%) did not have time pressure or receive feedback. The stress manipulation was found to be effective, as the reported state anxiety and visual analog scale on stress scores were higher for the stress group than for the non-stress group (both findings, *p* < 0.001). Consistent findings were found when accounting for trait anxiety as a moderator, with the exception of the state anxiety levels in high trait anxiety group. This stressing method combines the established stress conditions of uncontrollability (such as time pressures) and social evaluative threats (such as negative feedback). In addition, the method contains specific measures (such as a commitment statement and attention check questions) to enhance the internal validity by preventing and detecting cheating or random responses. This method can be deployed through any commonly available online software. It offers a simple and cost-effective way to collect data online – which fits the increasing need to carry out research in virtual and online environments.

**Supplementary Information:**

The online version contains supplementary material available at 10.3758/s13428-022-01915-3.

Generating stress in human subjects for research can be a challenging task (Ferreira, 2019). This is because, on the one hand, the experimental design needs to effectively generate stress but, on the other hand, avoid long-term effects on the participants (Ferreira, 2019). Adding to this challenge is the variability in how individuals perceive and react to the same stress factor (Epel et al., 2018; Lazarus & Folkman, 1984).

It has been observed that using only participants that can attend and participate in a study in person can have an impact on the diversity of the participant sample (Upadhyay & Lipkovich, 2020). Added to this, the value of being able to carry out online experiments has been highlighted particularly during the coronavirus pandemic (Wigginton et al., 2020) when much of the face-to-face research involving human subjects was paused worldwide. There has therefore been growing recognition of the value of creating opportunities for studies to be delivered online rather than face-to-face, including stress-inducing studies (Kirschbaum, 2021).

A meta-analysis of 208 laboratory-based stress studies found that the combination of social–evaluative threats (when one is judged negatively by others, such as receiving negative feedback) and uncontrollability (when nothing can be done to avoid negative consequences or change a situation, such as having a time limit for completing a task) were the stress factors that produce the greatest stress response in human subjects (Dickerson & Kemeny, 2004). Therefore, methods that combine social–evaluative threats and uncontrollability elements, such as the Trier Social Stress Test (TSST; Kirschbaum et al., 1993), considered the “gold standard” for inducing experimental stress in human subjects (Allen et al., 2017; Le et al., 2020), have potential for effectively inducing stress in an online setting.

Several studies have been conducted to try and validate online versions of TSST, delivered through virtual reality tools (e.g., Zimmer et al., 2019), and more recently delivered by video conferencing online (Eagle et al., 2021; Gunnar et al., 2021; Harvie et al., 2021). However, some of these Internet-delivered studies did not include a control group (Eagle et al., 2021; Gunnar et al., 2021), which limits the opportunity to understand and interpret the outcomes of the stress manipulation, for example, by not accounting for potential additional psychological stress as a result of video conferencing (Riedl, 2021). One study included a control group (Harvie et al., 2021), but required the (virtual) presence of at least three experimenters (i.e., the researcher and two panelists) in each video conferencing session, which limits online stress studies to live tasks in which the presence of the researchers is required nevertheless (virtually rather than in-person).

Therefore, in this study, alternative stressors were considered that combine social–evaluative threats and uncontrollability yet were still feasibly operationalized in an Internet-delivered environment without the need of the researchers to be present. One such stressor is the Trier Mental Challenge Test Stress Protocol originally developed by Kirschbaum et al. (1991)—referred to here as the ‘Mental Challenge Test’. In the Mental Challenge Test, participants are asked through programmed software to answer a number of arithmetic questions without a calculator under a time limit and receive feedback, such as “wrong” for incorrect answers (Kirschbaum et al., 1991). The studies that utilized the Mental Challenge Test were computer-assisted, yet, to date they have been conducted in the presence of the researchers (Allendorfer et al., 2014, 2019; Dedovic et al., 2005; Kirschbaum et al., 1991).

This study presents a method that has been developed for inducing stress in an online setting, without the presence of researchers (either in-person or virtually). This method may enable advancements in stress research, by accessing large number of international participants rapidly and in a cost-effective manner. In this method, participants were asked to answer a number of general knowledge and mathematical questions selected specifically for this study under stress conditions of social evaluative threats (such as displaying negative feedback) and uncontrollability (such as imposing time limits).

## Method

### Participants

Data were collected from 120 participants through the Prolific platform in a single session. Two participants in the stress group withdrew their data and were excluded from analysis. The final sample consisted of 118 participants, of whom *N* = 66, 56% were in the control group and *N* = 52, 44% in the stress group (see Table 1). Thirteen participants dropped out (*n* = 11 from the stress group and *n* = 2 from the control group). A drop-out is counted when a participant starts answering the mathematical and general knowledge questions then drops out by exiting the study.

**Table 1:** Demographical information of participants

	Mean (SD)	Range
Age	33.3 (7.0)	25–59
	*n*	Valid%
Sex		
Male	58	49.2
Female	60	50.8
Highest degree completed		
High school diploma/ A-levels or equivalent	18	15.3
Technical/ community college	9	7.6
Undergraduate degree (BA/BSc/Other)	46	39.0
Graduate degree (MA/MSc/MPhil/Other)	37	31.4
Doctorate degree (PhD/Other)	6	5.1
Other*	2	1.7

*The two participants reported PGCE (postgraduate certificate in education) as their highest completed education. Their data were coded within the ‘graduate degree’ holders, since PGCE is an advanced education after the bachelor’s degree

### Stress procedure

**Fig. 1:**
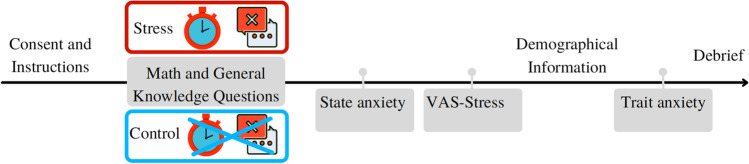
Graphic timeline of the experimental procedure

Participants signed the consent form and were then given instructions about the exercise (see Fig. 1). The consent form and instructions were carefully written to offer fully informed consent, but without revealing the specific aim of the study (i.e., inducing stress to participants). Then, participants were randomly allocated into either the stress or the control group through Qualtrics. The stress group was shown a warning message that performance was being monitored. They were then asked to answer a block of eight random mathematical/general knowledge questions with time limits and with feedback given (i.e., Stress Block A; see Appendices A, B and C for further details on the feedback messages and mathematical/general knowledge questions). If a participant answered a question incorrectly, a “  ” message in red would appear immediately on the screen. Conversely, a neutral “**OK**” message appeared in grey if a question was answered correctly. If the time allocated to the question ran out, a “  ” message appeared in red.

At the end of the mathematical/general knowledge question block, either a neutral message or a negative message was given to participants, depending on their performance (compared to a preset criterion score of three correct answers). If the participant scored three correct answers or lower in this block, then a negative message would appear explicitly comparing the individual score with those of other participants. This had the potential to further increase the social evaluative threat component of stress (Dickerson & Kemeny, 2004; Kirschbaum et al., 1991). If the participant scored four or more questions correctly in this block, a neutral message would appear that had no reference to individual or group performance. This approach was repeated in two more blocks (i.e., Stress Blocks B and C). The control group was asked to complete a comparable number and genre of questions but without feedback or a time limit. Questions were randomized through Qualtrics. To prevent and detect cheating or random responses, a range of quality assurance measures were included, such as adding a commitment statement, including a tool to detect potential bot responses and attention check questions (see Appendix A).

After three blocks of mathematical/ general knowledge questions, the participants were asked to complete the state anxiety scale (Spielberger et al., 1983) and a visual analogue scale on stress, referred to as ‘VAS-stress’ scale from here onwards. Next, participants were asked to provide their demographic information of age, sex, and their highest level of education. Participants were then asked to complete the trait anxiety scale (Spielberger et al., 1983). At the end of the experiment, participants were debriefed that this study specifically aimed to induce momentary stress. In the debrief, participants were given the opportunity to withdraw their data without giving a reason and without it affecting the rights and benefits (such as payment) to which they were entitled, or it having any negative repercussions for them.

### Stress manipulation check

The effectiveness of the stress manipulation was assessed and validated using two self-reported measures. First, to capture the situational anxiety levels of participants (i.e., the anxiety feelings in the present moment; see Appendix D), the state scale of the State–Trait Anxiety Inventory (STAI) was used (Spielberger et al., 1983). This state anxiety scale is a validated and commonly used measure for various stress manipulations (Arora et al., 2010; LeBlanc et al., 2005; Spielberger et al., 1983; Tanida et al., 2007). The scale consists of 20 statements (e.g., I feel nervous) for which users indicate their degree of agreement on a 4-point scale, in regard to how they feel ‘right now’ (score range is from 20 to 80; Spielberger et al., 1983). Second, following the approach of Le et al. (2020), participants were asked to report their stress levels on a VAS-stress, retrospectively: “Looking back, how stressed did you feel throughout answering the mathematical and general knowledge questions?” The participants rated their feelings from 0% (not stressed at all) to 100% (extremely stressed).

### Trait anxiety

Participants were also asked to complete the STAI trait anxiety scale (Spielberger et al., 1983; see Appendix E) to ensure that the background anxiety levels of participants do not confound the reported state anxiety or VAS-stress levels. The trait scale consists of 20 statements that measure how people ‘generally’ feel (score range from 20 to 80). The STAI manual recommends placing the trait anxiety scale, after the state anxiety scale if both scales are administered together, because the former measures a more stable anxiety construct that should not be affected with situational stress (Spielberger et al., 1983). Accordingly, the trait anxiety scale was placed at the end of the experiment.

## Results

### Overall stress and trait anxiety

The mean stress levels, as measured by the state anxiety scale, was significantly higher for the stress group (*M* = 48.89, *SD* = 13.01) than for the control group (*M* = 34.35, *SD* = 10.66), *M* = – 14.54, 95% CI [– 18.85, – 10.22], *t*(116) = – 6.67, *p* < 0.001, Cohen’s *d* = – 1.24. In addition, participants in the stress group (*M* = 73.17, *SD* = 24.01) reported higher VAS-stress ratings than the control group (*M* = 30.55, *SD* = 22.90). This was also a statistically significant difference, *M* = – 42.63, 95% CI [– 51.22, – 34.04], *t*(116) = – 9.83, *p* < 0.001, *d* = – 1.82. On average, the stress (*M* = 45.79, *SD* = 11.30) and non-stress groups (*M* = 41.58, *SD* = 12.37) were comparable in terms of their background stress (i.e., trait anxiety levels), *M* = – 4.21 , 95% CI [– 8.59, 0.16], *t*(116) = – 1.91, *p* = 0.059, *d* = – 0.35.

### Trait anxiety as a stress moderator

Two linear regression models were run to investigate whether the trait anxiety or the demographical variables (i.e., age, sex, and education) moderated the reported state anxiety or VAS-stress scores. In both models, the trait anxiety was the only factor (*p* < 0.001) that moderated the dependent variables. In addition, trait anxiety was significantly correlated with both state anxiety (*r*(118) = .55, *p* < 0.001) and VAS-stress scale (*r*(118) = .33, *p* < 0.001).

Hence, it was necessary to account for trait anxiety, as a background stress, to further understand the effectiveness of the online stressor presented here. To do so, participants were divided into three homogenous groups in terms of reported trait anxiety levels: low, moderate, and high anxiety (this approach is similar to Horikawa and Yagi (2012)). The high anxiety group (*N* = 35; *n* = 15 in the control condition and *n* = 20 in the stress condition) were those whose trait scores were 0.5 *SD* above the mean trait score of 43.43 (*SD* = 12.04). Conversely, the low anxiety group (*N* = 40; *n* = 27 in the control condition and *n* = 13 in the stress condition) were those whose trait scores were 0.5 *SD* below the mean trait score. The rest of participants (*N* = 43; *n* = 24 in the control condition and *n* = 19 in the stress condition) were classified to have moderate trait anxiety levels.

**Fig. 2:**
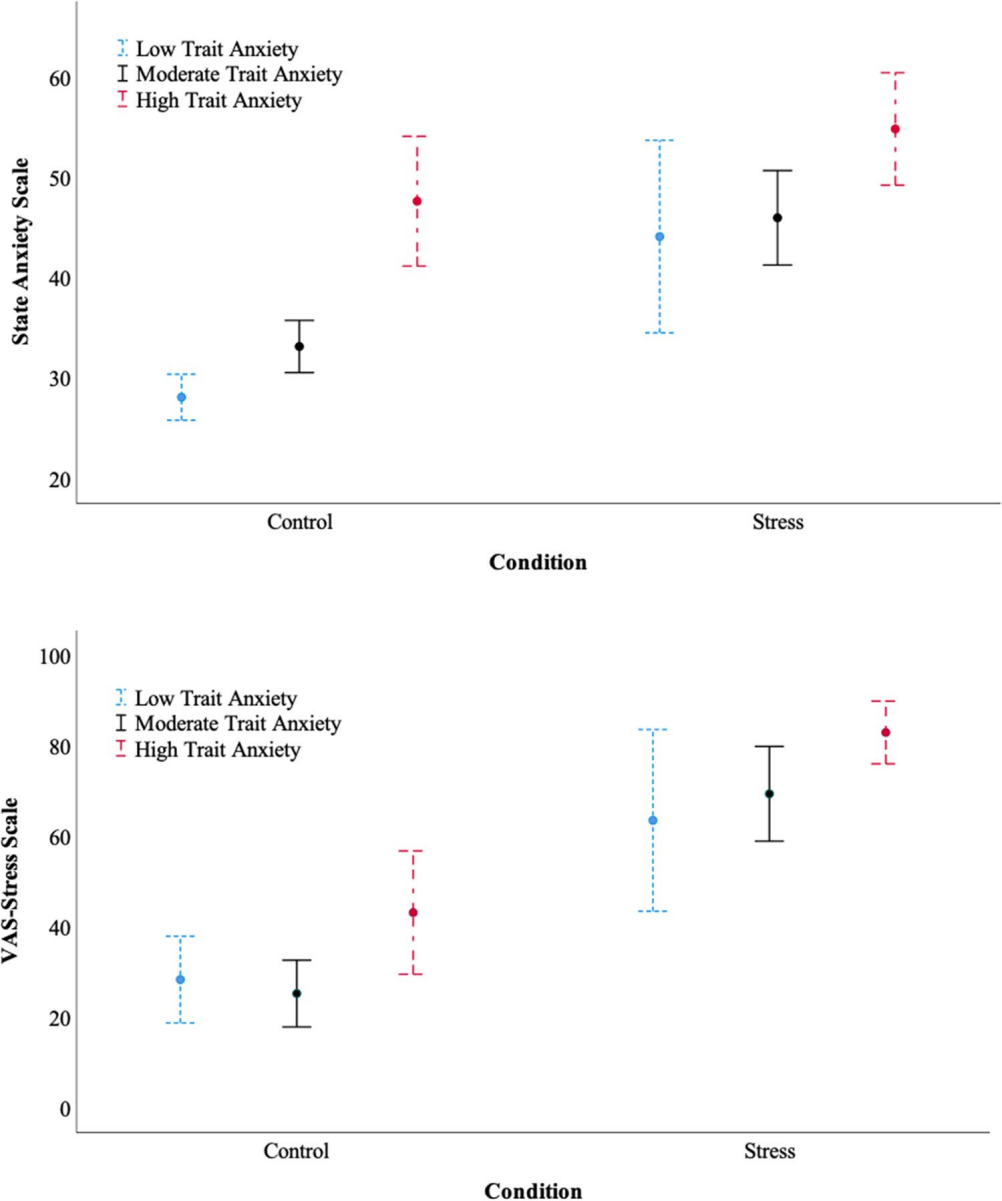
Mean state anxiety (top) and VAS-stress scores (bottom) for low, moderate, and high trait anxiety participant groups. Error bars reflect 95% confidence intervals

The state anxiety levels varied significantly between the stress and control conditions, in the low anxiety group (*M* = – 16.00, 95% CI [– 25.77, – 6.23], Welch’s *t*(13.57) = – 3.52, *p* = 0.004, *d* = – 1.19) and moderate anxiety group (*M* = – 12.82, 95% CI [– 17.75, – 7.90], *t*(41) = – 5.26, *p* < .001, *d* = – 1.61), but not in the high anxiety group (*M* = – 7.20, 95% CI [– 15.43, 1.03], *t*(33) = – 1.78, *p* = 0.084, *d* = – 0.61; Fig. 2). However, when comparing the VAS-stress scores, there were statistical significant differences in all the three anxiety groups (low anxiety: *M* = – 35.24, 95% CI [– 54.00, – 16.49], *t*(38) = – 3.80, *p* = 0.001, *d* = – 1.28; moderate anxiety: *M* = – 44.21, 95% CI [– 56.30, – 32.13], *t*(41) = – 7.39, *p* < 0.001, *d* = – 2.27; high anxiety: *M* = – 39.87, 95% CI [– 54.77, – 24.96], Welch’s *t*(21.48) = – 5.55, *p* < 0.001, *d* = – 1.90). Note that Welch’s *t* test is used when the assumption of homogeneity of variances has been violated, as assessed by Levene’s test for equality of variances.

### Performance on stress blocks

The majority (67.3–88.5%) of participants in the stress group scored three correct responses or less in stress blocks A, B, and C. This means that those participants received negative feedback after completing those blocks of questions. One participant was able to score 7 of 8 questions correctly in Block C, and no one scored 8 of 8 questions correctly (see Table 2).

**Table 2:** Frequency and cumulative percentages of correct responses in stress Blocks A, B, and C

Correct response	Stress Block A		Stress Block B		Stress Block C	
*N*	*N*	%	*N*	%	*N*	%
0	10	19.2	7	13.5	5	9.6
1	21	59.6	19	50.0	5	19.2
2	10	78.8	10	69.2	13	44.2
3	4	86.5	10	88.5	12	67.3
4	3	92.3	5	98.1	7	80.8
5	2	96.2	1	100	6	92.3
6	2	100	0	100	3	98.1
7	0	100	0	100	1	100
8	0	100	0	100	0	100

## Discussion

The stress manipulation was found to be effective in the sample who participated in this study. The state anxiety and VAS-stress scores were significantly higher for the stress group than the control group, with and without accounting for trait anxiety as a moderator. The exception was the state anxiety levels in the high trait anxiety group. Here, the state anxiety levels in the stress condition were still higher than the non-stress condition, although the difference was not statistically significant. One possible explanation is that the online stress method was not effective enough to induce momentary stress to already highly anxious participants—a clear sign of a ceiling effect.

Directly comparing our findings with published studies on stress-inducing methods can be limited (Narvaez Linares et al., 2020), especially that the online stressors are by their very nature less powerful than classical in-person stress tasks. Variations of TSST in previous research were able to cause elevations in state anxiety and VAS-stress levels comparable to the current stressor, but with smaller sample sizes. For instance, Guez et al. (2016) and Le et al. (2020) reported large effect sizes of their stressors on state anxiety (η^2^_p_ = 0.23, *N* = 46) and VAS on stress (*d* = 1.74, *N* = 76), respectively. This difference in magnitude is likely to be due to a number of factors that may include the absence of researchers during the stress-inducing period. Notably, however, our findings appear to be more in line with the impact of established stressors that had minimal interactions of investigators during the stress manipulation (Dedovic et al., 2005; see Discussion in p. 325).

The stress stimuli selected for this study appear to be challenging since most participants scored 3 or less questions correctly. Thus, the selected stress stimuli made it possible to give negative and potentially stressful feedback to participants in all three stress blocks. It may also be inferred from the data that engagement of some participants in answering the questions in the stress blocks may have been sustained (e.g., some participants were able to score four, five, six, or even seven questions correctly in a block, all of which were above the preset criterion score of three (see Table 2)). However, we cannot rule out the possibility that this procedure might lead to reduced engagement in some participants. Future studies should incorporate a consideration of whether low engagement/motivation might influence scores if, for example, a cognitive task was used after the stress induction.

The higher drop-out rate in the stress condition compared with the control condition could be due to a number of factors, namely the stress manipulation effectively causing stress and thus reduced motivation to complete the difficult tasks. The drop-out rate in this study appears to be higher than other validated stress methods. For instance, in a recent TSST method that was delivered by Zoom, one of 72 participants discontinued the study during the stress period (although it is worth noting that a total of 31 participants dropped out by the end of the experiment for other reasons, such as not showing up in scheduled sessions; Eagle et al., 2021).

Participants recruited through crowdsourcing platforms, as in the current study, appear to have a higher dropout rate than in-person/offline studies (Stewart et al., 2017; Zhou & Fishbach, 2016). This may be due a range of factors including participants having the ability to preview the study (Stewart et al., 2017), and potentially returning the study before completing the tasks and without affecting their reputation score on the crowdsourcing platforms (Palan & Schitter, 2018). Furthermore, there may be fewer barriers to dropping out of an online study due to the anonymity afforded by the online setting in comparison to dropping out of a live study (in person, or online but with a video connection with the researchers). In addition, researchers may not be aware of participants who have dropped out as they do not count towards the quota allocated in a crowdsourcing platform, and thus researchers under-report them in published papers (Zhou & Fishbach, 2016).

Importantly, drop-outs can be condition-dependent, for reasons such as experiencing more mental fatigue in one condition compared to the other (Zhou & Fishbach, 2016). Though selective attrition can potentially influence internal validity, we do not feel that this caused a meaningful impact on our findings, because the remaining randomized sample sizes in each condition for the method validation were reasonably comparable (i.e., 56% in comparison to 44%). Nevertheless, it may be beneficial for studies that use crowdsourcing platforms to include proactive countermeasure strategies (e.g., telling participants upfront that dropping out could affect the quality of data; Reips, 2000; Zhou & Fishbach, 2016).

A number of limitations do exist in regard to using this online stress method that should be addressed in future studies. First, the findings from this study are based on the assessment of stress from self-report measures (Nisbett & Wilson, 1977). Future research can include additional physiological measures, such as the approach taken by Harvie et al. (2021) who had participants measure their own heart rate.

Another limitation is that we did not balance the baseline stress (e.g., via VAS) for both groups. We were concerned that placing a VAS before the stress manipulation (so we could balance it across conditions) could impact feelings and expectations of the participants, and hence impact their performance (e.g., Christensen-Szalanski & Willham, 1991).

Furthermore, as with any remote online study, there is no control over what participants do during the exercise. Despite the effort made by the researchers to control experimental stimuli and set explicit instructions for the exercise, participants are not monitored and may be carrying out other activities while taking part in the study (such as doing the exercise while relaxing on the sofa compared to a desk). Such variations in behavior in completing the exercise may have the potential to influence the stress levels of participants, as opposed to being solely induced by the stress stimuli themselves.

Nevertheless, this is the first method that has been designed and used to induce stress in human participants effectively online without the presence of the researchers. It offers a cost-effective and easy-to-use method to induce momentary stress to human subjects in a controlled manner in an online setting. In addition, by not requiring the researchers to be agents of stress, the online method also enables quick access to large participant samples globally through crowdsourcing platforms (Peer et al., 2017). The method includes unpredictable social evaluative threats common in everyday life, including those in professional domains (e.g., Arora et al. 2010), which means it is a method that can offer a degree of ecological validity.

## Conclusions

This paper presents a new and ecologically valid method to stress human subjects in an online setting without the presence of researchers. This method offers a cost-effective way to collect data from a diverse range of participant cohorts, which is particularly useful in situations where there is a need to carry out research in online environments. The building blocks of this method (such as having specific measures to enhance data quality collected) could be useful for in a wide range of studies that aim to collect quality psychological data online.

## Supplementary Information

ESM 1 (DOCX 121 kb)

## Data Availability

The datasets generated and/or analyzed during the current study are available from the corresponding author on reasonable request. In addition, the programmed study link could be shared for current and/ or future research on reasonable request. None of the data or materials for the experiments reported here is available in a publicly accessible respiratory, and none of the experiments was preregistered.
