# Supplementary material for: A method to induce stress in human subjects in online research environments
Source: Behav Res Methods. 2022 Jul 25;55(5):2575–82. doi: 10.3758/s13428-022-01915-3 (PMC9311341; doi:10.3758/s13428-022-01915-3)
Supplement: Supplementary file 1 — (DOCX 121 kb) [file 13428_2022_1915_MOESM1_ESM.docx]

**Supplementary** **Materials**

## Appendix A: Development of the Online Stress Method

This study presents a method developed for inducing stress online, but without the presence of researchers. The development of this stress method—adapted from the Trier Mental Challenge Test Stress Protocol originally developed by Kirschbaum et al. (1991)—had three phases. In Phase I, stress stimuli that consisted of mathematical and general knowledge questions was selected. In Phase II, the stress stimuli were piloted to determine the mean response time for each question and to determine the accuracy rate for each question. In Phase III, the stress elements, such as calculating time deadlines for answering the questions based on the mean response times in Phase II, were identified.

## Phase I – Selection of Stress Stimuli

Two main categories of questions were selected for the online method: mathematical questions (*N* = 30) and general knowledge questions (*N* = 30). Because the study was conducted online and without the presence of researchers, it was critical to have a mix of questions for inducing stress. This way, participants were prevented from anticipating what they might be asked, to minimize the possibility of participants finding the answers through an online search, and as a mechanism for sustaining the motivation of the participants to complete the exercise (Yip 2004).

Three genres of mathematical questions were selected: “tricky” (10 questions), numerical reasoning (8 questions), and basic arithmetic (12 questions; see Table 1 for example questions and Appendix B in the Supplemental Materials for the full list of questions). The mathematics questions were carefully selected so that they would have varying levels of difficulties and could be solved mentally (i.e., without a calculator and without the use of a paper and pencil).

The ‘tricky’ genre consisted of questions that seem straightforward and intuitive but are rarely answered correctly (Gardner, 1986; Kahneman, 2011). The numerical reasoning questions required basic knowledge of mathematics and have been adapted from numerical reasoning tests used to test job applicants (Smith, 2017). These two genres of questions can be mentally challenging because they require more than merely a calculator (given that participants can have access to calculators). The third genre— the basic arithmetic questions— involves fundamental arithmetical operations, such as addition and multiplication, and resemble the mathematics questions used in the Mental Challenge Test (Allendorfer et al., 2019; Dedovic et al., 2005; Kirschbaum et al., 1991). To increase the difficulty level, no parentheses were added within the equations.

All the mathematical questions required free-text entry answers and the answers are intended to be whole numbers (without fractions) to minimize the opportunity for participant confusion and to simplify coding for the answers (for example, if fractions were used, then both 2.5 and 2 ^1^_/2_ would need to be coded as correct answers). Participants were instructed to input only the numerical answer for each question without a description (for example, “bananas” in question 7) or a unit (for example, “pounds” in question 18). A content validation was programmed for all the mathematical questions to ensure that participants only provided numeric answers.

The general knowledge questions were all multiple-choice questions derived from two sources. Sixteen questions came from a bank of questions used in professional workshops to which participants often provide wrong answers (see questions 31–46 in Appendix B). The other 14 questions were identified through an online web search for ‘easy general knowledge questions’ (see questions 47–60 in Appendix B) in order to include a set of general knowledge questions of varying difficulty.

## Phase II – Piloting the Stress Stimuli

### Overview

A pilot study was undertaken in which participants were asked to answer all the selected questions in Phase I with no time limit and no feedback. The pilot study had two aims. First to assess the mean response time of each question in order to calculate a preset time deadline for the stress group. The second to select questions to allocate for the stress and control conditions of the experiment: the stress group was to be provided with questions that were often answered incorrectly. This would increase the likelihood of the stress group receiving negative feedback (such as “wrong”).

### Recruitment of Participants

The participants were recruited using the Prolific crowdsourcing platform widely used for academic research (Clemmow et al., 2020; Palan & Schitter, 2018; Peer et al., 2017). The participant selection criteria were 25–60 years of age with a minimum level of high school (or equivalent) education. The online experiments were developed and deployed using Qualtrics.

**Table 1**

Examples of Mathematical and General Knowledge Questions

| **Genre** | **Question** | **Answer** | **Source** |
| --- | --- | --- | --- |
| ‘Tricky’ Math  Questions | Q.1. A pen and pad cost one dollar and ten cents. The pen costs one dollar more than the pad. How much does the pad cost (in cents)? (*please type the number only*). | 5 cents | (Kahneman, 2011) |
|  | Q.5 A person was born on May 6, 30 B.C. He died on May 6, 30 A.D. How old was he when he died? (*please type the number only*). | 59 years | (Gardner, 1986) |
| Numerical Reasoning | Q12. The Arsenal football club “games won” to “games lost” record last season was 2:3. How many games did they play last season if all the games were either won or lost and Arsenal won 6 games? (*please type the number only*). | 15 games | (Smith, 2017) |
|  | Q15. A plant grows by 5% each year. Its height was 90 cm when it was planted. In which year will the plant exceed 1 m in height. (*please type the number only*). | Year 3 | (Smith, 2017) |
| Basic Arithmetic | Q19. What is the answer?  $3\times4-9=$ | 3 | These questions follow the paradigm of the Mental Challenge Stress Protocol (Kirschbaum et al., 1991). |
|  | Q28. What is the answer?  $4\times3^{2}\div1^{4}+4$= | 40 |  |
| General Knowledge | Q32. Which ocean goes to the deepest depths?   1. Pacific 2. Arctic 3. Atlantic 4. Indian 5. Southern | 1. Pacific | Taken from a bank of questions that have already been used in professional workshops. |
|  | Q49. Which planet is known as the Red Planet?   1. Neptune 2. Jupiter 3. Mars 4. Mercury 5. Earth | 1. Mars | Found in Edsys (2020) by searching the internet for “easy general knowledge questions” |

### Strategy for Response Quality

Participants recruited from online crowdsourcing platforms generally have high-quality responses and low “cheating” rates (Berinsky et al., 2012; Clifford & Jerit, 2016; Motta et al., 2016). For example, it has been identified that students were more inclined to look up the answers in web-based political knowledge surveys than crowdsourced users of Mechanical Turk, potentially due to crowdsourced participants having financial incentives to finish quickly rather than spending time looking up answers (Clifford & Jerit, 2016). As subsequent phases in developing the online stress method presented here depended on the responses from this pilot study, measures were included to prevent and detect low-quality responses. These measures aimed to improve the internal validity by preventing and detecting cheating or random responses.

#### **Commitment Statement**

Clifford and Jerit (2016) compared different methods for reducing cheating in online surveys (such as directly asking participants not to cheat or asking them to willingly commit to not use outside help). They found that a commitment request to participants with “yes” and “no” answers resulted in the lowest degrees of cheating and the greatest predictive validity. In addition, a commitment statement can avoid the unpredicted disadvantage regarding participants’ goodwill, such as when asking the participants directly not to cheat. Hence, a commitment statement using language adapted from Clifford and Jerit (2016) was used as a prevention measure to cheating:

*Commitment Statement:* Data of this scientific study may ultimately contribute to improving the decision-making in forensic science. Hence, it is important to us that you do NOT use outside sources, like a calculator or the internet. Will you answer the following questions without help from outside sources? (Yes / No)

#### **Question Wording Alternations**

Previous research suggested that “Googling cheaters” may look for factual answers online while completing the survey (Jensen & Thomsen, 2014). Such behavior is prevented or reduced in face-to-face surveys (Heerwegh & Loosveldt, 2008). Yet, because this study was conducted completely online, a new solution that has the potential to minimize online search activities was warranted. The solution utilized for this study entailed simple alterations to the question wording. Specifically, nonessential information or words in the question (such as names or dates) were amended, when possible, whereas the essential information was retained to increase the difficulty of looking for answers through an internet search.

In the example in Table 2, the name and title “Larygitis, a Greek orator” were nonessential to answering the question; hence, they were removed from the original question. Moreover, the day and month of birth were amended. The answer would still be the same—59 years old. In this example, when copying the original question for an online search, the question is found in the first search line as well as the source of the original question, but the amended question and its source do not appear in the entire first page of the online search.

Amending the nonessential information in the original questions was only done for the numeric reasoning and tricky questions. It was not possible to amend the words in the general knowledge questions because these questions did not typically contain nonessential information.

**Table 2**

*Example for Amending the Nonessential Words of Tricky Question Number 5*

| **Original Question** | **Amended Question** |
| --- | --- |
| Larygitis, a Greek orator, was born on July 4, 30 B.C. He died on July 4, 30 A.D. How old was he when he died? | A person was born on May 6, 30 B.C. He died on May 6, 30 A.D. How old was he when he died? |

#### **Image Not Text**

Researchers in the political science literature have discussed that image-based knowledge questions on individuals—as opposed to text-only questions—can be a useful way to minimize looking up answers online (Motta et al., 2017; Prior, 2014). Taking this approach, in the current study, the basic arithmetic questions were coded in Qualtrics as images, not text. This prevention measure did not allow participants to simply copy and paste the formulas online to find the answers.

#### **Randomization of the Questions**

Evidence suggests that some crowdsourced users may share experimental tasks on unofficial online forums where they can interact with other users and provide information on specific tasks (Schmidt, 2015). Randomization of questions has been suggested as a potential way to reduce cheating through collaborations with others (McLeod et al., 2003) and can help in minimizing the order effect (Oldendick 2008). Therefore in this method, the order of questions was randomized using Qualtrics.

#### **High Approval Rates**

Prolific offers a prescreening tool called “approval rate” for participants, which is the number of studies approved by researchers divided by the number of total studies completed by the participant. Therefore, the approval rate can be an indicative measure regarding the quality of data a Prolific participant may offer. It has been found that restricting participation to participants with high approval rates (specifically, above 95% approval ratings) is an effective method to enhance data quality (Peer et al., 2014). Therefore, for this study, only participants with a high approval rate of 95% or above were chosen.

#### **Attention Check Questions**

Attention check screeners are questions that are typically used to check whether participants read the instructions. They are embedded within the exercise and look similar to experimental stimuli in terms of length and format (such as the number of multiple-choice options). However, the answers may not follow a normal format or expectations, so participants will need to read the instructions to answer them (Oppenheimer et al., 2009; see Table 3). Some researchers have argued that attention checks may affect the responses of subsequent questions because they are in essence ‘trick questions’ (Kane & Barabas, 2019; Hauser et al., 2018).

However, given the nature of the subject pool (see Peer et al. (2017) on attention check failures from croudsourced participants), they are still being used in empirical research as useful tools to detect participant attentiveness to the study tasks (Clemmow et al., 2020). In addition, some researchers recommend using more than one attention check question to improve data quality (Berinsky et al., 2014). Therefore, two questions were used for Phase II to minimize potential impact, if any, on the overuse of attention checks to the responses of actual stress stimuli (see Table 3). One attention question resembled a general knowledge question, and one attention check question resembled a numerical reasoning/tricky question. An attention check question resembling basic arithmetic can be easily detected by the participants so no attention check question that looked similar to the basic mathematical questions was included.

**Table 3**

*Attention Check Questions*

| **Attention Check Question** | **Answer** | **Notes** |
| --- | --- | --- |
| What color is the sky? Please select “purple” as an answer to make sure you are paying attention.   1. White 2. Blue 3. Green 4. Purple 5. Black | Purple | Resembles general knowledge questions. |
| There are seven orange trees on a farm. These trees double in number every 6 months. Please type “orange” as an answer to this question to make sure that you are paying attention. What is the answer? | Orange | Resembles numerical reasoning and tricky questions. |

#### **Explicit Instructions**

Providing clear instructions can enhance the understanding of the experimental tasks by reducing ambiguity which can enhance the quality of responses (Alekseev et al., 2017). In this method, the language of the instructions was carefully written to minimise ambiguity regarding the tasks the participants were being asked to complete (e.g., the instructions made it clear that the use of a calculator and paper/pencil were not allowed):

*Instructions:* Please read the instructions carefully before proceeding:

1. You are asked to answer a number of questions to the best of your ability.
2. The questions in the study may include general knowledge questions, numerical reasoning questions, and so on.
3. The study should take approximately 45 min but not more than 60 min.
4. The use of a calculator is NOT permitted.
5. The use of a paper and pencil is NOT permitted.
6. Please rely on your own personal knowledge WITHOUT using internet searches.
7. You are asked to be complete the study in a single time session.
8. Please switch off distractions (like emails) and find a quiet place to focus on this study.
9. Please only take part in this study if you are using a desktop/laptop computer.

#### **Bot Reponses**

Computer programs may be used in crowdsourcing platforms to complete the tasks automatically, known as a bot response (Chmielewski & Kucker, 2020). Some researchers recommend using Captcha Verification (or Completely Automated Public Turing Test to tell Computers and Humans Apart) to ensure that the responses come from real human participants, not programs (Chmielewski & Kucker, 2020). For this study, one captcha verification question from Qualtrics was included at the beginning of the survey. The participants were presented with a challenge consisting of an image of words or characters that they needed to correctly complete before proceeding.

#### **Elimination of Low-Quality Responses**

The responses of all Prolific participants were accepted, and the participants were paid for their time. However, the plan was to carefully check the data for each participant considering multiple factors to decide whether or not to keep their data for the analysis (see Table 4).

The specific criteria outlined in Table 4 helped “red-flag” potential low-quality responses. Red-flagged responses were investigated thoroughly to determine whether there was a consistent pattern of low-quality response across different questions. Thus, the aim of the elimination criteria was not to provide quantifiable measures to eliminate low-quality responses or cheaters ( Berinsky et al., 2014), but to gain a more holistic view of the quality of responses for each participant.

**Table 4**

*Criteria for Eliminating Low-Quality Data From Analysis*

| **Attention check** | - The participant fails more than one attention check question. Evidence suggests that elimination of data based on a single screener may result in bias in research (Berinsky et al., 2014). |
| --- | --- |
| **Length of time** | - Consistently very high response times. That is, response times are repetitively much higher than those of other participants across multiple mathematical/general knowledge questions. Such response behaviors raise concerns that that they search for answers on the internet. For instance, Clifford and Jerit (2016) found that self-reported cheaters spent significantly more time in answering the general knowledge questions than non-cheaters. - Consistently very low response times. This response behavior raises concerns that participants randomly answer the questions, without cognitive effort (Börger, 2016). |
| **Pattern of subjects’ responses** | - High number of correct answers on difficult questions (i.e., more than just chance). - Consistently choosing the same responses. For example, choosing the same option in multiple choice questions (Clemmow et al., 2020). |

### Results of Phase II

#### **Quality of Responses**

In total, 35 participants were recruited via Prolific. The responses of five participants were excluded from the analysis, leaving a final sample of 30 participants (60% male, *n* = 18; age range = 25–47 years; *M* = 33.53 years, *SD* = 6.43 years). Data of the five participants were excluded due to their responses raising concerns; they showed a consistent pattern of low-quality responses based on the length of time they spent on the questions. The responses of two participants were consistently very fast, raising concerns over the cognitive effort made in answering the questions (Börger, 2016). The response times were even too short for potentially reading a question and thinking about the answer (for example, one participant spent 2.2 s, 2.7 s, and 2.8 s on questions 45, 41, and 42).

Conversely, the responses of the other three participants were consistently extremely slow with the response times of those participants being very high for a number of simple general knowledge questions (for example one participant spent around 3 min to answer question 49 on the longest river in the world). This increased response time raised concerns that these participants could be looking up answers via the web (Clifford & Jerit, 2016) and so they were excluded.

Only one participant failed one of the two attention check screeners. After careful assessment of the quality of the responses from that participant (see Table 4), their data were not excluded so that data were not eliminated based on a single screener as this may result in bias (Berinsky et al., 2014; Clemmow et al., 2020).

#### **Elimination of Outliers**

To calculate reasonable time limits for answering the questions, two established methods were compared. The first method sets a deadline by subtracting one standard deviation from the mean response time (Benson & Beach, 1996; Tsiga et al., 2013). The second method uses 70% of the established mean response times ( Kellogg et al., 1999). It is critical that established mean response times are not inflated as having outliers can potentially make the means (or the standard deviations) higher or lower than they should be. For example, if the standard deviation method was applied for the current study without any data treatment, then some of the deadlines would be too short (for example, a 1- or 2-s deadline) or would be negative (when the standard deviation is higher than the mean). Thus, in this study the outliers were excluded.

Ratcliff (1993) developed simulations of response times to test the different methods used in removing response time outliers based on the central tendency approach—a common method of response time data treatment (Whelan, 2008). Therefore in this study response-time outliers were eliminated following Ratcliff (1993):

1. Cutoffs should be chosen as a function of proportions of responses eliminated. Based on the reaction time data simulations, it was recommended that a reasonable range to choose cutoffs should aim to eliminate 5–15% of data (i.e., keep the central 85–95% of the data). In the current study, only 7.6% response time data points were excluded. This exclusion rate falls within the lower end of the recommended range.
2. When there is a high variability among the subjects’ response time means, as in the current study, then an elimination method using a standard deviation cutoff is recommended (Ratcliff, 1993). One of the cutoffs tested was 1.5 absolute standard deviation above the mean, which yielded acceptable results. This value (i.e., absolute z score = 1.5 *SD* or higher) was chosen for the current experiment because, upon preliminary assessment, this cutoff value allows for excluding 5–15% of the data (see recommendation 1, above).

In summary, 136 response time data points were at, or above, 1.5 standard deviation from the mean and thus were excluded (7.6%, *N* = 1,800). If the response times were considered an outlier using the method above, then, both the reaction times and the actual responses to the question were eliminated.

#### **Accuracy and Mean Response Times**

Appendix B in the Supplemental Materials show the accuracies and mean response times of all questions tested (*N* = 60). Accuracy (%) refers to the number of correct responses by number of total responses (i.e., *N* after removing outliers). The questions were divided into either the stress condition (*N* = 30) or the control condition (*N* = 30) based on accuracy. Stress questions were of the lowest accuracy for each type of question (see questions in bold in Appendix B).

## Phase III – Inclusion of Stress Elements

After the stress stimuli were tested in the pilot study, the following elements were included in the stress method:

### Time Limits

Having time limits to answer the question increases the sense of uncontrollability (Allen et al., 2017; Dickerson & Kemeny, 2004). There are different approaches to setting time limits such as having a fixed deadline for the participants to answer a block of mathematical questions (Kirschbaum et al., 1991) or using a program that continuously increases/decreases time limits depending on the subject’s performance (Dedovic et al., 2005). For this study, each question had a precalculated time limit that was the same across participants, regardless of performance. Using this approach, the participants would not be able to predict the deadlines for subsequent questions, potentially increasing the sense of uncontrollability and stress levels.

On assessing the two methods to set the time allowed for answering each question, a 70% mean method was more appropriate than a standard deviation method, as this latter method produced timeframes for a number of questions that were too short to read the question properly let alone answer it, even after eliminating the outliers. For example, in question 39, the deadline would be 3s using the standard deviation method and 9s using the 70% of mean method (see Appendix C in Supplemental Materials). If time pressures were too tight, the participants could have decided to randomly answer the general knowledge and mathematical questions, thus invalidating the stress stimuli. Time limits were rounded to the nearest whole number.

### Distribution of Stress Stimuli

The distribution of the mathematical/general knowledge questions in the stress and the control conditions was carefully considered. Forty-eight questions of the 60 questions used in the pilot study were selected with 12 questions being removed to ensure that the exercise was not too long and to ensure an even distribution of questions across the two groups (see Appendix C). For the even distribution, both groups were asked to answer the same number of questions (*N* = 24) and the same type of questions (see Table 5). Specifically, participants would be asked to answer eight questions in each block. When possible, questions that were comparable in terms of similarity of content for the stress and control group were used. For example, questions 16 and 11 were chosen for the control group and stress group, respectively, because they both involve distance or speed vector mathematical problems.

**Table 5**

*Even Distribution of 48 Mathematical/General Knowledge Questions Across Three Blocks*

| **Block** | **Question Type** | **Control Group** | **Stress Group** |
| --- | --- | --- | --- |
| Block A | Numerical Reasoning | 1 | 1 |
|  | Tricky Questions | 1 | 1 |
|  | Basic Arithmetic | 2 | 2 |
|  | General Knowledge Questions | 4 | 4 |
|  | **Total Questions** | **8** | **8** |
| Block B | Numerical Reasoning | 1 | 1 |
|  | Tricky Questions | 1 | 1 |
|  | Basic Arithmetic | 2 | 2 |
|  | General Knowledge Questions | 4 | 4 |
|  | **Total Questions** | **8** | **8** |
| Block C | Numerical Reasoning | 1 | 1 |
|  | Tricky Questions | 1 | 1 |
|  | Basic Arithmetic | 2 | 2 |
|  | General Knowledge Questions | 4 | 4 |
|  | **Total Questions** | **8** | **8** |

This approach was repeated in three blocks: eight questions in block A, eight questions in block B, and eight questions in block C. Block A was expected to generate feelings of stress in the participants, whereas blocks B and C were expected to restore the stress level so that it would not dissipate with time. After block C, the stress levels were reported (i.e., the stress manipulation check).

### Feedback

The feedback given to the participants is an important element in affecting the feelings of a social evaluative threat and the stress levels in participants (Allen et al., 2017; Dickerson & Kemeny, 2004). Incorporating negative feedback (such as receiving a message of “WRONG”) has been identified as a social–evaluative threat element that leads to elevated levels of stress (Dedovic et al., 2005). This study adapted similar feedback messages to those used in previous studies that utilized the Trier Mental Challenge Test (Allendorfer et al., 2014, 2019; Dedovic et al., 2005; Kirschbaum et al., 1991).

#### **Feedback Before the Stress Stimuli Block**

Feedback or messages were shown to participants at three stages: before, during, and after each stress stimuli block (see Table 6). Before the first block of mathematical/general knowledge questions, participants in the control group were told that their performance was not being monitored. This message was expected to reduce stress, thus increasing the difference in stress levels between the stress and nonstress groups.

**Table 6**

*Feedback Given to Participants Before, During and After Each Stress Stimuli Block*

| **Feedback** | **Stress Group** | **Control Group** |
| --- | --- | --- |
| Before Stress Stimuli Block | **“WARNING! PLEASE READ CAREFULLY:**   - You are reminded that your performance is being monitored by Prolific Academic. - You are reminded that there is a required minimum performance when answering the questions. Your individual performance will be compared with the rest of the participants to determine whether your data will be used in the study. - *Note that some questions have a limited amount of time for you to answer them, whereas others don’t. The questions with the limited amount time will have a clock showing the remaining time.*”   Participants click on the following message to continue the study: “I understand that my performance is being monitored.” | “Note that your performance is NOT being evaluated, so please attempt to answer the questions as accurately as possible.”  Participants click on the following message to continue the study: “I understand that my performance is NOT being monitored.” |
| During Stress Stimuli Block | **WRONG!** for incorrect answer.  **TIME OUT!** when the allocated time runs out.  **OK** for correct answers. | No feedback given. |
| After Stress Stimuli Block | If the participant scores three correct answers or lower, the following message will appear: **Unfortunately, your individual score for the math and general knowledge questions you have just completed was lower than, or did not supersede, the average performance of participants.**  If the participant scores four correct answers or higher, the following message will appear: **OK, you have completed this block of math and general knowledge questions, and you can now proceed to the next step.** | No feedback given. |

Conversely, participants in the stress group were warned that their performance was being monitored by multiple stakeholders—Prolific and the researchers. In addition, it was clarified that a minimum individual performance was required and that their individual performance would be compared with that of other participants for inclusion in the study using statements adapted and modified from Dedovic et al. (2005). This was expected to increase the feelings of social evaluative threat and thus stress levels of the participants. Another element of this framing was that participants may have felt the risk of losing their monetary incentives from Prolific, which would potentially increase their stress levels (even though the consent form clearly stated that the participants would be paid for their time, even if they wished to withdraw their data). These statements were not deceptive, as opposed to some previous stress research studies (such as Allendorfer et al., 2014, 2019) but were provided within the context of Prolific stating that it monitors response quality to avoid bot-like responses (Bradley, 2018).

#### **Feedback During the Stress Stimuli Block**

During the stress block, if a participant answered a question incorrectly, a “**WRONG!**” message in red would appear immediately on the screen. Conversely, a neutral “**OK**” message appeared in grey if the question were answered correctly (see Kirschbaum et al. (1991)). While some studies have utilized positive feedback, such as “CORRECT” (Dedovic et al., 2005), neutral feedback was deemed to be more suitable in this study because it was expected to result in a greater difference in stress levels between the control and stress groups. If the time allocated to the question ran out, a “**TIME OUT!**” message appeared in red (i.e., the participant left the space empty or did not select any option in multiple choice questions). For the control condition, no feedback was given.

For this exercise, the color red was used for negative messages (such as “**WRONG!**”) and the original Qualtrics color of grey was used for neutral messages (such as “**OK”**). This color-coding was used for the feedback messages before, during, and after the stress stimuli blocks as the color red is associated with negative emotions, such as anger and rage (Joosten et al., 2012; Plutchik, 2001), which can be related to stress levels (Du et al., 2018; Kutchma, 2003).

#### **Feedback After the Stress Stimuli Block**

After a block of eight questions, the nonstress group received no feedback messages. However, the stress group received one of two messages, as shown in Table 6. If the participant scored three correct answers or lower in this block, then a negative message would appear explicitly comparing the individual score with those of other participants. This had the potential to further increase the social evaluative threat component of stress (Dickerson & Kemeny, 2004; Kirschbaum et al., 1991). If the participant scored four or more questions correct in this block, a neutral message would appear that had no reference to individual or group performance.

Previous studies that took place with the researchers being present have provided feedback after the stress stimuli in different ways including increasing or decreasing the difficulty of questions to enforce a result of 20–45% correct answers with participants being told that the average performance was 80–90% (Dedovic et al. 2005). Other approaches have included asking participants to write their scores on a blackboard in front of the group (Kirschbaum et al., 1991). However, because the exercise in this study took place completely online and without the presence of the researchers, a different approach was needed. It was necessary to choose a fixed cut-off average performance for each block of mathematical/general knowledge questions. Hence, the questions were distributed across the three blocks in the stress condition so that their average accuracy percentages were comparable (i.e., 35.9% to 36.9%; see Appendix C).

There were eight questions in each block and all the questions had the same weight, so that there can be a consistent grade level requirement in each block. Individual score can be automatically calculated via Qualtrics by adding the number of correct responses. As a result, there were nine possible individual scores for each block (Table 7) and it was possible to approximate the *average performance* for all three blocks (i.e., 35.9% to 36.9%) to 37.5%. Therefore, the average score performance within each block of eight questions was considered as 37.5% (or three correct answers).

**Table 7**

*Possible Individual Scores for Each Stress Block*

| **Number of Correct Responses** | 0 | 1 | 2 | 3 | 4 | 5 | 6 | 7 | 8 |
| --- | --- | --- | --- | --- | --- | --- | --- | --- | --- |
| **Individual Accuracy Rate** | 0% | 12.5% | 25% | 37.5% | 50% | 62.5% | 75% | 87.5% | 100% |

**References**

Alekseev, A., Charness, G., & Gneezy, U. (2017). Experimental methods: When and why contextual instructions are important. *Journal of Economic Behavior & Organization*, *134*, 48–59. https://doi.org/10.1016/j.jebo.2016.12.005

Allen, A. P., Kennedy, P. J., Dockray, S., Cryan, J. F., Dinan, T. G., & Clarke, G. (2017). The Trier Social Stress Test: Principles and practice. *Neurobiology of Stress*, *6*, 113–126. https://doi.org/10.1016/j.ynstr.2016.11.001

Allendorfer, J. B., Heyse, H., Mendoza, L., Nelson, E. B., Eliassen, J. C., Storrs, J. M., & Szaflarski, J. P. (2014). Physiologic and cortical response to acute psychosocial stress in left temporal lobe epilepsy—A pilot cross-sectional fMRI study. *Epilepsy & Behavior*, *36*, 115–123. https://doi.org/10.1016/j.yebeh.2014.05.003

Allendorfer, J. B., Nenert, R., Hernando, K. A., DeWolfe, J. L., Pati, S., Thomas, A. E., Billeaud, N., Martin, R. C., & Szaflarski, J. P. (2019). FMRI response to acute psychological stress differentiates patients with psychogenic non-epileptic seizures from healthy controls – A biochemical and neuroimaging biomarker study. *NeuroImage: Clinical*, *24*, 101967. https://doi.org/10.1016/j.nicl.2019.101967

Benson III, L., & Beach, L. R. (1996). The Effects of Time Constraints on the Prechoice Screening of Decision Options. *Organizational Behavior and Human Decision Processes*, *67*(2), 222–228. https://doi.org/10.1006/obhd.1996.0075

Berinsky, A. J., Huber, G. A., & Lenz, G. S. (2012). Evaluating online labor markets for experimental research: Amazon.com’s Mechanical Turk. *Political Analysis*, *20*(3), 351–368. https://doi.org/10.1093/pan/mpr057

Berinsky, A. J., Margolis, M. F., & Sances, M. W. (2014). Separating the shirkers from the workers? Making sure respondents pay attention on self-administered surveys. *American Journal of Political Science*, *58*(3), 739–753. https://doi.org/10.1111/ajps.12081

Börger, T. (2016). Are fast responses more random? Testing the effect of response time on scale in an online choice experiment. *Environmental and Resource Economics*, *65*(2), 389–413. https://doi.org/10.1007/s10640-015-9905-1

Bradley, P. (2018). *Bots and data quality on crowdsourcing platforms*. Prolific Academic. https://blog.prolific.co/bots-and-data-quality-on-crowdsourcing-platforms/, accessed 20 May 2020.

Chmielewski, M., & Kucker, S. C. (2020). An MTurk crisis? Shifts in data quality and the impact on study results. *Social Psychological and Personality Science*, *11*(4), 464–473. https://doi.org/10.1177/1948550619875149

Clemmow, C., Schumann, S., Salman, N. L., & Gill, P. (2020). The base rate study: Developing base rates for risk factors and indicators for engagement in violent extremism. *Journal of Forensic Sciences*, *65*(3), 865–881. https://doi.org/10.1111/1556-4029.14282

Clifford, S., & Jerit, J. (2016). Cheating on political knowledge questions in online surveys: An assessment of the problem and solutions. *Public Opinion Quarterly*, *80*(4), 858–887. https://doi.org/10.1093/poq/nfw030

Dedovic, K., Renwick, R., Mahani, N. K., Engert, V., Lupien, S. J., & Pruessner, J. C. (2005). The Montreal Imaging Stress Task: Using functional imaging to investigate the effects of perceiving and processing psychosocial stress in the human brain. *Journal of Psychiatry & Neuroscience*, *30*(5), 319–325.

Dickerson, S. S., & Kemeny, M. E. (2004). Acute stressors and cortisol responses: A theoretical integration and synthesis of laboratory research. *Psychological Bulletin*, *130*(3), 355–391. https://doi.org/10.1037/0033-2909.130.3.355

Du, J., Huang, J., An, Y., & Xu, W. (2018). The relationship between stress and negative emotion: The Mediating role of rumination. *Clinical Research and Trials*, *4*(1). https://doi.org/10.15761/CRT.1000208

edsys. (2020). *General knowledge for kids*. https://www.edsys.in/general-knowledge-for-kids-105-questions-and-answers/, accessed 20 May 2020.

Gardner, M. (1986). *Entertaining mathematical puzzles*. http://www.arvindguptatoys.com/arvindgupta/martingardnerone.pdf, accessed 20 May 2020.

Heerwegh, D., & Loosveldt, G. (2008). Face-to-face versus web surveying in a high-internet-coverage population: Differences in response quality. *Public Opinion Quarterly*, *72*(5), 836–846. https://doi.org/10.1093/poq/nfn045

Jensen, C., & Thomsen, J. P. F. (2014). Self-reported cheating in web surveys on political knowledge. *Quality & Quantity*, *48*(6), 3343–3354. https://doi.org/10.1007/s11135-013-9960-z

Joosten, E., van Lankveld, G., & Spronck, P. (2012). Influencing player emotions using colors. *Journal of Intelligent Computing*, *3*(2), 76–86.

Kahneman, D. (2011). *Thinking fast and slow*. Penguin Random House UK.

Kellogg, J. S., Hopko, D. R., & Ashcraft, M. H. (1999). The effects of time pressure on arithmetic performance. *Journal of Anxiety Disorders*, *13*(6), 591–600. https://doi.org/10.1016/S0887-6185(99)00025-0

Kirschbaum, C., Diedrich, O., Gehrke, J., Wüst, S., & Hellhammer, D. H. (1991). Cortisol and behavior: The “Trier Mental Challenge Test” (TMCT)—First evaluation of a new psychological stress test. In A. Ehlers, W. Fiegenbaum, I. Florin, & J. Margraf (Eds.), *Perspectives and promises of clinical psychology* (pp. 67–78). Springer.

Kutchma, T. M. (2003). *The Effects of Room Color on Stress Perception: Red versus Green Environments*. *3*, 12.

McLeod, I., Zhang, Y., & Yu, H. (2003). Multiple-Choice Randomization. *Journal of Statistics Education*, *11*(1), 4. https://doi.org/10.1080/10691898.2003.11910695

Motta, M. P., Callaghan, T. H., & Smith, B. (2017). Looking for answers: Identifying search behavior and improving knowledge-based data quality in online surveys. *International Journal of Public Opinion Research*, *29*(4), 575–603. https://doi.org/10.1093/ijpor/edw027

Oppenheimer, D. M., Meyvis, T., & Davidenko, N. (2009). Instructional manipulation checks: Detecting satisficing to increase statistical power. *Journal of Experimental Social Psychology*, *45*(4), 867–872. https://doi.org/10.1016/j.jesp.2009.03.009

Palan, S., & Schitter, C. (2018). Prolific.ac—A subject pool for online experiments. *Journal of Behavioral and Experimental Finance*, *17*, 22–27. https://doi.org/10.1016/j.jbef.2017.12.004

Peer, E., Brandimarte, L., Samat, S., & Acquisti, A. (2017). Beyond the Turk: Alternative platforms for crowdsourcing behavioral research. *Journal of Experimental Social Psychology*, *70*, 153–163. https://doi.org/10.1016/j.jesp.2017.01.006

Peer, E., Vosgerau, J., & Acquisti, A. (2014). Reputation as a sufficient condition for data quality on Amazon Mechanical Turk. *Behavior Research Methods*, *46*(4), 1023–1031. https://doi.org/10.3758/s13428-013-0434-y

Plutchik, R. (2001). The Nature of Emotions: Human emotions have deep evolutionary roots, a fact that may explain their complexity and provide tools for clinical practice. *American Scientist*, *89*(4), 344–350.

Prior, M. (2014). Visual Political Knowledge: A Different Road to Competence? *The Journal of Politics*, *76*(1), 41–57. https://doi.org/10.1017/S0022381613001096

Ratcliff, R. (1993). Methods for dealing with reaction time outliers. *Psychological Bulletin*, *114*(3), 510–532. https://doi.org/10.1037/0033-2909.114.3.510

Schmidt, G. B. (2015). Fifty Days an MTurk Worker: The Social and Motivational Context for Amazon Mechanical Turk Workers. *Industrial and Organizational Psychology*, *8*(2), 165–171. https://doi.org/10.1017/iop.2015.20

Smith, H. (2017). *How to pass numerical reasoning tests* (3rd ed.). Kogan Page Limited.

Tsiga, E., Panagopoulou, E., Sevdalis, N., Montgomery, A., & Benos, A. (2013). The influence of time pressure on adherence to guidelines in primary care: An experimental study. *BMJ Open*, *3*(4), e002700. https://doi.org/10.1136/bmjopen-2013-002700

Whelan, R. (2008). Effective analysis of reaction time data. *The Psychological Record*, *58*(3), 475–482. https://doi.org/10.1007/BF03395630

Yip, D. Y. (2004). Questioning skills for conceptual change in science instruction. *Journal of Biological Education*, *38*(2), 76–83. https://doi.org/10.1080/00219266.2004.9655905

### Appendix B: Accuracy and Mean Response Times for Each Question Selected

**Table B1**

*Tricky Mathematical Questions (Numeric Text-Entry). Half the questions with the lower accuracy rates than the other half are in bold.*

| **Question** | **Answer** | ***N*** | **Accuracy (%)** | **Mean (secs)** | ***SD* (secs)** |
| --- | --- | --- | --- | --- | --- |
| 1. **A pen and pad cost one dollar and ten cents. The pen costs one dollar more than the pad. How much does the pad cost (in cents)? (*please type the number only*).** | **5 cents** | **27** | **33.3** | **41.238** | **34.969** |
| 1. There is a patch of flowers in a garden. Every day, the patch doubles in size. If it takes 48 days for the patch to cover the entire garden, how many days would it take for the patch to cover half of the garden? (*please type the number only*). | 47 days | 29 | 44.8 | 33.788 | 22.227 |
| 1. **What is the answer?**   $\boldsymbol{6\div2(1+2)}\mathbf{=}$ | **9** | **29** | **27.6** | **23.951** | **12.500** |
| 1. In a juice factory, it takes 5 machines 5 seconds to produce 5 boxes of juice. How many seconds would it take 100 machines to make 100 boxes of juice? (*please type the number only*). | 5 seconds | 27 | 55.6 | 34.105 | 23.791 |
| 1. **A person was born on May 6, 30 B.C. He died on May 6, 30 A.D. How old was he when he died? (*please type the number only*).** | **59 years** | **29** | **0.0** | **32.839** | **24.286** |
| 1. **Divide 30 by ½ and add 10. What is the result?** | **70** | **28** | **28.6** | **13.604** | **6.406** |
| 1. A boy had five bananas and ate all but three. How many bananas were left? (*please type the number only*) | 3 bananas | 28 | 78.6 | 15.034 | 6.598 |
| 1. What two whole numbers—not fractions—make the unlucky number 13 when multiplied together? (*you may type the two numbers in any order as ‘number 1 x number 2’*). | 1 x 13 | 27 | 74.0 | 49.818 | 24.908 |
| 1. If there are 12 one-cent stamps in a dozen, then how many two-cent stamps are in a dozen? (*please type the number only*). | 12 stamps | 29 | 48.3 | 21.022 | 8.716 |
| 1. **If a clock takes five seconds to strike 6 o’clock, how long will it take to strike 12 o’clock? (*please type the number only*).** | **11 seconds** | **27** | **0.0** | **30.491** | **17.226** |

**Table B2**

*Numerical Reasoning Questions (Numeric Text-Entry). Half the questions with the lower accuracy rates than the other half are in bold.*

| **Question** | **Answer** | ***N*** | **Accuracy (%)** | **Mean (secs)** | ***SD* (secs)** |
| --- | --- | --- | --- | --- | --- |
| 1. **Ruth goes walking to work at 8.10am. She stops to buy a tea and read her newspaper for 15 minutes and arrives at work at 9.55am. The distance between her house and work is 6 miles. What is her average walking speed in miles per hour? (*please type the number only*).** | **4 miles per hour** | **28** | **50.0** | **79.835** | **37.960** |
| 1. **Arsenal football club ‘games won’ to ‘games lost’ record last season was 2 : 3. How many games did they play last season if all the games were either won or lost and Arsenal won 6 games? (*please type the number only*).** | **15 games** | **26** | **46.2** | **58.236** | **28.477** |
| 1. The total entrance price for a theatre show for 2 adults and 2 children is $24. The ticket price for an adult is twice the price for a child’s ticket. How much does an adult’s ticket cost? (*please type the number only*). | $8 | 28 | 75.0 | 61.711 | 38.434 |
| 1. **While on vacation in Italy, William withdraws €200 from his bank account and receives a pile of €10 and €20 notes. How many €10 notes does William receive if he receives 14 notes in total? (*please type the number only*).** | **8 notes** | **26** | **65.4** | **91.728** | **51.193** |
| 1. **A plant grows by 5% each year. Its height was 90 cm when it was planted. In which year will the plant exceed 1 m in height? (*please type the number only*).** | **Year 3** | **28** | **39.3** | **45.878** | **24.254** |
| 1. A car travels along a road at a rate of 40 mph for 4.5 hours; how far does the car travel in miles? (*please type the number only*). | 180 miles | 28 | 75.0 | 36.905 | 20.130 |
| 1. The price of a barrel of oil increased from £20 to £24 between May and August 1993. By what percentage did the price of oil increase during this period? (*please type the number only*). | 20% | 27 | 66.7 | 41.406 | 29.452 |
| 1. A rooster and a hen together weigh 27 pounds. If the rooster weighs twice as much as the hen does, how much does the hen weighs in pounds? (*please type the number only*). | 9 pounds | 27 | 77.8 | 42.773 | 21.990 |

**Table B3**

*Basic Arithmetic (Numeric Text-Entry). Half the questions with the lower accuracy rates than the other half are in bold.*

| **Question** | **Answer** | ***N*** | **Accuracy (%)** | **Mean (secs)** | ***SD* (secs)** |
| --- | --- | --- | --- | --- | --- |
| 1. What is the answer?   $3\times4-9=$ | 3 | 28 | 100.0 | 8.645 | 3.238 |
| 1. **What is the answer?**   $\boldsymbol{2-6\div3=}$ | **0** | **26** | **88.5** | **10.585** | **5.585** |
| 1. **What is the answer?**   $\boldsymbol{8\div4+2 \times5=}$ | **12** | **29** | **65.5** | **16.324** | **6.890** |
| 1. What is the answer?   $93-16=$ | 77 | 26 | 96.2 | 14.621 | 6.796 |
| 1. What is the answer?   $84- 19-7=$ | 58 | 28 | 96.4 | 22.104 | 8.713 |
| 1. **What is the answer?**   $\boldsymbol{3\times3-2\times2=}$ | **5** | **29** | **58.6** | **14.997** | **11.297** |
| 1. What is the answer?   $75-8=$ | 67 | 26 | 96.2 | 9.197 | 3.139 |
| 1. **What is the answer?**   $\boldsymbol{20-5+2\times3=}$ | **21** | **28** | **57.1** | **19.347** | **9.466** |
| 1. What is the answer?   $5 \times2^{2}-3$= | 17 | 27 | 88.9 | 11.754 | 6.028 |
| 1. **What is the answer?**   $\boldsymbol{4\times}\boldsymbol{3}^{\boldsymbol{2}}\boldsymbol{\div}\boldsymbol{1}^{\boldsymbol{4}}\boldsymbol{+4}$**=** | **40** | **26** | **61.5** | **27.165** | **13.569** |
| 1. **What is the answer?**   $\boldsymbol{3-3\times3\div}\boldsymbol{3}^{\boldsymbol{2}}$**=** | **2** | **27** | **44.4** | **24.921** | **19.548** |
| 1. What is the answer?   $12\div6\times3\div2$= | 3 | 28 | 89.3 | 22.511 | 16.945 |

**Table B4**

*General Knowledge Questions (Multiple Choice). Half the questions with the lower accuracy rates than the other half are in bold.*

| **Question** | **Answer** | ***N*** | **Accuracy (%)** | **Mean (secs)** | ***SD* (secs)** |
| --- | --- | --- | --- | --- | --- |
| 1. In the following sentence, there are two missing words. Which two words from the options (A–E) below fit the missing words best:   The higher court’s reversal of its previous ruling on the issue of suspected terrorists                                  its reputation for                             .   1. sustained, infallibility 2. compromised, consistency 3. bolstered, doggedness 4. aggravated, inflexibility 5. dispelled, vacillation | B | 26 | 76.9 | 47.599 | 19.616 |
| 1. Which ocean goes to the deepest depths? 2. Pacific 3. Arctic 4. Atlantic 5. Indian 6. Southern | A | 27 | 59.3 | 10.212 | 6.118 |
| 1. **What is the meaning of the musical term ‘allegro’?** 2. **loud** 3. **soft** 4. **quick** 5. **slow** 6. **stop** | **C** | **28** | **57.1** | **13.062** | **6.789** |
| 1. **If** $\frac{\boldsymbol{x}}{\boldsymbol{3}}$ **=** $\boldsymbol{x}^{\boldsymbol{2}}$ **, then value of** $\boldsymbol{x}$ **can be which of the following? I. -1/3; II. 0; III. 1/3** 2. **I only** 3. **II only** 4. **III only** 5. **II and III only** 6. **I, II, and III** | **D** | **28** | **25.0** | **45.234** | **30.067** |
| 1. **If the population of a town doubles every 10 years, then population in the year (X + 100) years will be how many times the population in the year (X)?** 2. **512** 3. **100** 4. **1,024** 5. **10** 6. **1,000** | **C** | **29** | **24.1** | **65.090** | **37.891** |
| 1. **Which one of the following categories is NOT awarded a Nobel Prize?** 2. **Physics** 3. **Chemistry** 4. **Biology** 5. **Medicine** 6. **Literature** | **C** | **28** | **39.3** | **20.515** | **14.047** |
| 1. **What percentage cocoa solids must chocolate contain to be legally called chocolate?** 2. **99** 3. **50** 4. **15** 5. **25** 6. **35** | **E** | **29** | **20.7** | **15.811** | **10.073** |
| 1. **What is the major vitamin found in brown rice?**   **A. A**  **B. B**  **C. C**  **D. D**  **E. E** | **B** | **29** | **34.5** | **16.835** | **11.059** |
| 1. **Samite is a type of?** 2. **Cake** 3. **Stone** 4. **Dog** 5. **Fabric** 6. **Horse** | **D** | **29** | **17.2** | **12.369** | **9.612** |
| 1. **Who wrote ‘don't count your chickens before they are hatched’?** 2. **Shakespeare** 3. **Ben Franklin** 4. **Chaucer** 5. **Aesop** 6. **Dickens** | **D** | **27** | **22.2** | **10.356** | **5.499** |
| 1. **What is the capital of Cambodia?** 2. **Luang Prabang** 3. **Vientiane** 4. **Ho Chi Minh** 5. **Phnom Penh** 6. **Hebei** | **D** | **28** | **39.3** | **13.525** | **5.830** |
| 1. **What is a shooting star?** 2. **Dying star** 3. **Meteor** 4. **Comet** 5. **Asteroid** 6. **Supernova** | **B** | **26** | **34.6** | **12.308** | **4.548** |
| 1. **What percent of people live north of the equator?** 2. **70%** 3. **75%** 4. **80%** 5. **85%** 6. **90%** | **E** | **28** | **3.6** | **13.472** | **5.060** |
| 1. **Which one of these is not an insect?** 2. **Flea** 3. **Tick** 4. **Mosquito** 5. **Beetle** 6. **Butterfly** | **B** | **28** | **21.4** | **19.246** | **9.324** |
| 1. **What will you get if you shake whipping cream in a glass can for 10 minutes?** 2. **Whipped Cream** 3. **Cheese** 4. **Milk** 5. **Butter** 6. **Yogurt** | **D** | **28** | **21.4** | **20.748** | **11.353** |
| 1. What animal first reached Earth’s orbit alive? 2. Mice 3. Ape 4. Cockroach 5. Cat 6. Dog | E | 27 | 63.0 | 9.343 | 3.292 |
| 1. What is the official World Health Organization abbreviation for the current global pandemic?   A. COVID-19  B. Coronavirus  C. SARS-CoV-2  D. 2019-nCoV  E. SARS-CoV | A | 29 | 65.5 | 14.810 | 7.558 |
| 1. How many days are in a year?   A. 151  B. 243  C. 365  D. 411  E. 502 | C | 29 | 100.0 | 4.835 | 1.769 |
| 1. **Which is the longest river on earth?**   **A. Nile**  **B. Amazon**  **C. Congo**  **D. Lena**  **E. Mekong** | **A** | **26** | **50.0** | **7.829** | **4.337** |
| 1. Which is the tallest mountain in the world?   A. Fuji  B. Mount Kilimanjaro  C. Table Mountain  D. Mont Blanc  E. Mount Everest | E | 29 | 86.2 | 8.520 | 4.056 |
| 1. Where is Kenya located?   A. Asia  B. Africa  C. Europe  D. South America  E. Australia | B | 29 | 100.0 | 5.175 | 2.144 |
| 1. Which planet is known as the Red Planet?   A. Neptune  B. Jupiter  C. Mars  D. Mercury  E. Earth | C | 26 | 96.2 | 5.278 | 2.551 |
| 1. Which animal is known as the ‘Ship of the Desert?’   A. Fox  B. Camel  C. Lizard  D. Whale  E. Scorpion | B | 29 | 100.0 | 10.090 | 4.935 |
| 1. Who was the first man to walk on the moon?   A. Cristiano Ronaldo  B. Captain Cook  C. Neil Armstrong  D. Ibn Battuta  E. Laika | C | 28 | 100.0 | 6.316 | 2.318 |
| 1. **Which is the most spoken language in the world?**   **A. English**  **B. Spanish**  **C. Arabic**  **D. French**  **E. Chinese** | **E** | **26** | **34.6** | **6.497** | **2.640** |
| 1. What is the capital of the United States?   A. Washington, DC  B. New York City  C. Los Angeles  D. Chicago  E. Miami | A | 29 | 100.0 | 5.923 | 4.357 |
| 1. Who is the founder of Microsoft?   A. Donald Trump  B. Bill Gates  C. Mark Zuckerberg  D. Steve Jobs  E. Elon Musk | B | 27 | 96.3 | 4.939 | 2.280 |
| 1. Global warming is caused by the excess of which type of gas?   A. Oxygen  B. Nitrogen  C. Argon  D. Carbon dioxide  E. Carbon monoxide | D | 28 | 78.6 | 12.225 | 6.414 |
| 1. Which country is home to the kangaroo?   A. Australia  B. Congo  C. Angola  D. Zambia  E. New Zealand | A | 29 | 100.0 | 5.682 | 2.652 |
| 1. Who invented the telephone?   A. Thomas Edison  B. Wright Brothers  C. Guglielmo Marconi  D. Leonardo da Vinci  E. Alexander Graham Bell | E | 29 | 75.9 | 12.117 | 11.289 |

**Appendix C: Order of Questions Within the Blocks**

Specific mathematical/general knowledge questions for each of the three blocks are provided below; accuracy percentages of each question from the baseline study and the average percentage accuracy for each block are included. The precalculated deadlines of two methods are shown, namely the mean minus one standard deviation and 70% of the mean.

| **Block** | **Question Type** | **Control Group** | **Stress Group** | | | |
| --- | --- | --- | --- | --- | --- | --- |
|  |  | **Selected question** | **Selected question** | **Baseline accuracy**  **(%)** | **Deadline (secs)** | |
|  |  |  |  |  | **Mean minus SD** | **70% of Mean** |
| A | Numerical Reasoning | Q16. A car travels along a road at a rate of 40 mph for 4.5 hours. How far does the car travel in miles? (*please type the number only*). | Q11. Ruth goes walking to work at 8.10 a.m. She stops to buy a tea and read her newspaper for 15 minutes and arrives at work at 9.55 a.m. The distance between her house and work is 6 miles. What is her average walking speed in miles per hour? (*please type the number only*). | 50.0 | 42 | 56 |
|  | Tricky Questions | Q7. A boy had five bananas and ate all but three. How many bananas were left? (*please type the number only*) | Q1. A pen and pad cost one dollar and ten cents. The pen costs one dollar more than the pad. How much does the pad cost (in cents)? (*please type the number only*). | 33.3 | 6 | 29 |
|  | Basic Arithmetic | Q25. What is the answer?  $75-8=$ | Q21. What is the answer?  $8\div4+2 \times5=$ | 65.5 | 9 | 11 |
|  |  | Q27. What is the answer?  $5 \times2^{2}-3$= | Q29. What is the answer?  $3-3\times3\div3^{2}$= | 44.4 | 5 | 17 |
|  | General Knowledge Questions | Q56. What is the capital of the United States?  A. Washington, DC  B. New York City  C. Los Angeles  D. Chicago  E. Miami | Q34. If $\frac{\boldsymbol{x}}{\boldsymbol{3}}$ **=** $\boldsymbol{x}^{\boldsymbol{2}}$ , then value of $\boldsymbol{x}$ can be which of the following? I. -1/3; II. 0; III. 1/3   1. I only 2. II only 3. III only 4. II and III only 5. I, II, and III | 25.0 | 15 | 32 |
|  |  | Q60. Who invented the telephone?  A. Thomas Edison  B. Wright Brothers  C. Guglielmo Marconi  D. Leonardo da Vinci  E. Alexander Graham Bell | Q36. Which one of the following categories is NOT awarded a Nobel Prize?   1. Physics 2. Chemistry 3. Biology 4. Medicine 5. Literature | 39.3 | 6 | 14 |
|  |  | Q51. Where is Kenya located?  A. Asia  B. Africa  C. Europe  D. South America  E. Australia | Q37. What percentage cocoa solids must chocolate contain to be legally called chocolate?   1. 99 2. 50 3. 15 4. 25 5. 35 | 20.7 | 6 | 11 |
|  |  | Q59. Which country is home to the kangaroo?  A. Australia  B. Congo  C. Angola  D. Zambia  E. New Zealand | Q39. Samite is a type of?   1. Cake 2. Stone 3. Dog 4. Fabric 5. Horse | 17.2 | 3 | 9 |
|  |  |  | **Average Score for Stress Group** | **36.9** | **-** | **-** |
| B | Numerical Reasoning | Q18. A rooster and a hen together weigh 27 pounds. If the rooster weighs twice as much as the hen does, how much does the hen weighs in pounds? (*please type the number only*). | Q12. The Arsenal football club “games won” to “games lost” record last season was 2:3. How many games did they play last season if all the games were either won or lost and Arsenal won 6 games? (*please type the number only*). | 46.2 | 30 | 41 |
|  | Tricky Questions | Q4. In a juice factory, it takes 5 machines 5 seconds to produce 5 boxes of juice. How many seconds would it take 100 machines to make 100 boxes of juice? (*please type the number only*). | Q6. Divide 30 by ½ and add 10. What is the result? | 28.6 | 7 | 10 |
|  | Basic Arithmetic | Q22. What is the answer?  $93-16=$ | Q20. What is the answer?  $2-6\div3=$ | 88.5 | 5 | 7 |
|  |  | Q30. What is the answer?  $12\div6\times3\div2$= | Q28. What is the answer?  $4\times3^{2}\div1^{4}+4$= | 61.5 | 14 | 19 |
|  | General Knowledge Questions | Q31. In the following sentence, there are two missing words; which two words from the options (A–E) below fit the missing words best:  The higher court’s reversal of its previous ruling on the issue of suspected terrorists                                  its reputation for                             .   1. sustained, infallibility 2. compromised, consistency 3. bolstered, doggedness 4. aggravated, inflexibility 5. dispelled, vacillation | Q43. What percent of people live north of the equator?   1. 70% 2. 75% 3. 80% 4. 85% 5. 90% | 3.6 | 8 | 9 |
|  |  | Q57. Who is the founder of Microsoft?  A. Donald Trump  B. Bill Gates  C. Mark Zuckerberg  D. Steve Jobs  E. Elon Musk | Q40. Who wrote “don’t count your chickens before they are hatched”?   1. Shakespeare 2. Ben Franklin 3. Chaucer 4. Aesop 5. Dickens | 22.2 | 5 | 7 |
|  |  | Q53. Which animal is known as the “Ship of the Desert?”  A. Fox  B. Camel  C. Lizard  D. Whale  E. Scorpion | Q44. Which one of these is not an insect?   1. Flea 2. Tick 3. Mosquito 4. Beetle 5. Butterfly | 21.4 | 10 | 13 |
|  |  | Q54. Who was the first man to walk on the moon?  A. Cristiano Ronaldo  B. Captain Cook  C. Neil Armstrong  D. Ibn Battuta  E. Laika | Q45. What will you get if you shake whipping cream in a glass can for 10 minutes?   1. Whipped cream 2. Cheese 3. Milk 4. Butter 5. Yogurt | 21.4 | 9 | 15 |
|  |  |  | **Average Score for Stress Group** | **36.7** | **-** | **-** |
| C | Numerical Reasoning | Q13. The total entrance price for a theatre show for 2 adults and 2 children is $24. The ticket price for an adult is twice the price for a child’s ticket. How much does an adult’s ticket cost? (*please type the number only*). | Q15. A plant grows by 5% each year. Its height was 90 cm when it was planted. In which year will the plant exceed 1 m in height. (*please type the number only*). | 39.3 | 22 | 32 |
|  | Tricky Questions | Q9. If there are 12 one-cent stamps in a dozen, then how many two-cent stamps are in a dozen? (*please type the number only*). | Q5. A person was born on May 6, 30 B.C. He died on May 6, 30 A.D. How old was he when he died? (*please type the number only*). | 0.0 | 9 | 23 |
|  | Basic Arithmetic | Q19. What is the answer?  $3\times4-9=$ | Q26. What is the answer?  $20-5+2\times3=$ | 57.1 | 10 | 14 |
|  |  | Q23. What is the answer?  $84- 19-7=$ | Q24. What is the answer?  $3\times3-2\times2=$ | 58.6 | 4 | 10 |
|  | General Knowledge Questions | Q48. How many days are in a year?  A. 151  B. 243  C. 365  D. 411  E. 502 | Q35. If the population of a town doubles every 10 years, then population in the year (X + 100) years will be how many times the population in the year (X)?   1. 512 2. 100 3. 1,024 4. 10 5. 1,000 | 24.1 | 27 | 46 |
|  |  | Q58. Global warming is caused by the excess of which type of gas?  A. Oxygen  B. Nitrogen  C. Argon  D. Carbon dioxide  E. Carbon monoxide | Q38. What is the major vitamin found in brown rice?  A. A  B. B  C. C  D. D  E. E | 34.5 | 6 | 12 |
|  |  | Q52. Which planet is known as the Red Planet?  A. Neptune  B. Jupiter  C. Mars  D. Mercury  E. Earth | Q42. What is a shooting star?   1. Dying star 2. Meteor 3. Comet 4. Asteroid 5. Supernova | 34.6 | 8 | 9 |
|  |  | Q50. Which is the tallest mountain in the world?  A. Fuji  B. Mount Kilimanjaro  C. Table Mountain  D. Mont Blanc  E. Mount Everest | Q41. What is the capital of Cambodia?  A. Luang Prabang  B. Vientiane  C. Ho Chi Minh  D. Phnom Penh  E. Hebei | 39.3 | 8 | 9 |
|  |  |  | **Average Score for Stress Group** | **35.9** | **-** | **-** |

### Appendix D: Self-Evaluation Questionnaire (PART A)

A number of statements that people have used to describe themselves are given below. Read each statement and then choose the response to indicate how you feel *right now*, that is, *at the moment*. There are no right or wrong answers. Do not spend too much time on any one statement but give the answer that seems to describe your feelings best. Note that item 13 is an additional item was added to the questionnaire as an attention check screener.

|  |  | **Not at all** | **Somewhat** | **Moderately so** | **Very much so** |
| --- | --- | --- | --- | --- | --- |
| 1 | I feel calm | 1 | 2 | 3 | 4 |
| 2 | I feel secure | 1 | 2 | 3 | 4 |
| 3 | I am tense | 1 | 2 | 3 | 4 |
| 4 | I feel strained | 1 | 2 | 3 | 4 |
| 5 | I feel at ease | 1 | 2 | 3 | 4 |
| 6 | I feel upset | 1 | 2 | 3 | 4 |
| 7 | I am presently  worrying over  possible misfortunes | 1 | 2 | 3 | 4 |
| 8 | I feel satisfied | 1 | 2 | 3 | 4 |
| 9 | I feel frightened | 1 | 2 | 3 | 4 |
| 10 | I feel comfortable | 1 | 2 | 3 | 4 |
| 11 | I feel self-confident | 1 | 2 | 3 | 4 |
| 12 | I feel nervous | 1 | 2 | 3 | 4 |
| 13 | Please tick “somewhat” | 1 | 2 | 3 | 4 |
| 14 | I am jittery |  |  |  |  |
| 15 | I feel indecisive | 1 | 2 | 3 | 4 |
| 16 | I am relaxed | 1 | 2 | 3 | 4 |
| 17 | I feel content | 1 | 2 | 3 | 4 |
| 18 | I am worried | 1 | 2 | 3 | 4 |
| 19 | I feel confused | 1 | 2 | 3 | 4 |
| 20 | I feel steady | 1 | 2 | 3 | 4 |
| 21 | I feel pleasant | 1 | 2 | 3 | 4 |

### Appendix E: Self-Evaluation Questionnaire (PART B)

A number of statements which people have used to describe themselves are given below. Read each statement and then choose the response to indicate how you *generally feel*. Note that PART B of the questionnaire is different from PART A that you completed earlier. In part A, it was about how you feel right now, but here, in part B, it is how you generally feel as a person, NOT how you specifically feel at this moment.

|  |  | **Almost Never** | **Sometimes** | **Often** | **Almost Always** |
| --- | --- | --- | --- | --- | --- |
| 1 | I feel pleasant | 1 | 2 | 3 | 4 |
| 2 | I feel nervous and restless | 1 | 2 | 3 | 4 |
| 3 | I feel satisfied with myself | 1 | 2 | 3 | 4 |
| 4 | I wish I could be as happy as others seem to be | 1 | 2 | 3 | 4 |
| 5 | I feel like a failure | 1 | 2 | 3 | 4 |
| 6 | I feel rested | 1 | 2 | 3 | 4 |
| 7 | I feel “calm, cool, and collected” | 1 | 2 | 3 | 4 |
| 8 | I feel that difficulties are piling up so that I cannot overcome them | 1 | 2 | 3 | 4 |
| 9 | I worry too much over something that really doesn’t matter | 1 | 2 | 3 | 4 |
| 10 | I am happy | 1 | 2 | 3 | 4 |
| 11 | I have disturbing thoughts | 1 | 2 | 3 | 4 |
| 12 | I lack self-confidence | 1 | 2 | 3 | 4 |
| 13 | Please tick “often” | 1 | 2 | 3 | 4 |
| 14 | I feel secure |  |  |  |  |
| 15 | I make decisions easily | 1 | 2 | 3 | 4 |
| 16 | I feel inadequate | 1 | 2 | 3 | 4 |
| 17 | I am content | 1 | 2 | 3 | 4 |
| 18 | Some unimportant thought runs through my mind and bothers me | 1 | 2 | 3 | 4 |
| 19 | I take disappointments so keenly that I can’t put them out of my mind | 1 | 2 | 3 | 4 |
| 20 | I am a steady person | 1 | 2 | 3 | 4 |
| 21 | I get in a state of tension or turmoil as I think over my recent concerns and interests | 1 | 2 | 3 | 4 |
